# Transport Stress Changes Blood Biochemistry, Antioxidant Defense System, and Hepatic HSPs mRNA Expressions of Channel Catfish *Ictalurus punctatus*

**DOI:** 10.3389/fphys.2018.01628

**Published:** 2018-11-20

**Authors:** Mohamed M. Refaey, Dapeng Li

**Affiliations:** ^1^College of Fisheries, Hubei Provincial Engineering Laboratory for Pond Aquaculture, National Demonstration Center for Experimental Aquaculture Education, Huazhong Agricultural University, Wuhan, China; ^2^Department of Animal Production, Faculty of Agriculture, Mansoura University, Al-Mansoura, Egypt

**Keywords:** transport, heat shock proteins, antioxidative status, stress, channel catfish, fish welfare

## Abstract

Transport procedures usually cause fish stress. The purpose of this study was to investigate the effect of transport stress on blood biochemical profiles, oxidative stress biomarkers, and hepatic heat shock proteins (HSPs) of channel catfish (*Ictalurus punctatus*). Fish (body weight 55.57 ± 5.13 g) were randomly distributed to two groups, the control, and the treatment. The control group was kept under the normal culture conditions. The treatment group was exposed to the process of transport (3.5 h). Fish samples were collected before transport, after packing and at 0, 1, 6, 24, 72, and 168 h after transport, respectively. Transport caused a significant increase in the serum concentrations of cortisol, glucose, total cholesterol, and triglyceride, as well as, the activity of aspartate aminotransferase at 0 and 1 h after transport compared with non-transported fish and the basal level. Blood total protein content significantly declined in the transported fish. Total antioxidant capacity (T-AOC), malonaldehyde content, and the activities of both glutathione peroxidase and catalase significantly increased in fish within 6 h after transport. The transported fish exhibited a significant higher level in either the concentration of nitric oxide or the mRNA expressions of both hepatic HSP70 and HSP90. It is concluded that transport triggers stress response of *I. punctatus*, leading to the obvious change in antioxidant capacity. *I. punctatus* need to be more care after transport to recover from transport stress.

## Introduction

Transport of live fish is an inevitable procedure in aquaculture system. Transport process including several procedures whether pre-transport procedures (such as collection, grading, netting, air exposure, and packing) and during transport process procedures (such as water movement, vibrations, and water condition change), which are stressful to fish (Paterson et al., 2003; Dhanasiri et al., 2013; Pakhira et al., 2015). The stress resulted from transport often causes fish growth suppression, higher mortality (Akinrotimi, 2006), and the susceptibility to diseases (Maule et al., 1989), and the increment of rearing costs (Gomes et al., 2006). Consequently, transport procedure should be designed to minimize stress (Dobšikova et al., 2009). Additionally, numerous fish species respond to transport stress by lifting the contents of circulating catecholamines and corticosteroids such as cortisol hormone that is considered a biomarker of the primary stress response (Barton and Iwama, 1991). The elevation of cortisol level is followed by many secondary responses like elevated blood glucose content and altered electrolyte homeostasis (Barton and Iwama, 1991; McDonald and Milligan, 1997), and metabolic alterations as hyperglycaemia, hyperlactaemia, and hypercholesterolaemia (Mommsen et al., 1999). The extent of the responses is dependent on the intensity of the stress (Sumpter, 1997).

Stress triggers irregular oxidative responses in the aerobic metabolic pathways, which induces the formation reactive oxygen species (ROS). Accumulation of ROS inside cells leads to oxidative stress (OS), resulting in lipid peroxidation, protein carboxylation, and damage of the nucleic acid (Halliwell and Gutteridge, 2001). Many stressors e.g., hypoxia, hyperoxia, increased ammonia, and high stocking density, can cause oxidative stress (Lushchak, 2011; Sahin et al., 2014; Sun et al., 2014). Fish have several enzymes such as glutathione peroxidase (GSH-PX) and catalase (CAT), working as defenses system against OS (Trenzado et al., 2009). Total antioxidant capacity (T-AOC) is a general indicator of antioxidant defense against free radical, which is used to assay the level of oxidative stress in organisms (O'Brien et al., 2000). Moreover, GSH-PX is worked on protection of membranes against the oxidative degradation of lipids (Ritola et al., 2002). Nitric oxide (NO) is one of the smallest a bioactive cellular component (Gong et al., 2004), which involves several physiological functions such as protecting from oxidative and hypoxia stress, homeostasis, cytotoxicity (Agnisola, 2005).

On the cellular level, stress stimulates another physiological response through increased synthesis of heat shock proteins (HSPs). HSPs include a large group of conserved proteins that classified according to their molecular weight (Basu et al., 2002). HSPs regulate of cellular protein structure and acting as housekeeping and cytoprotective functions (Pockley, 2003), as well as, it work as companion to the metabolic activities of both protein and lipid (Roberts et al., 2010). Additionally, HSPs play an important role in a cellular homeostasis under abrupt environmental fluctuations (Iwama et al., 1998). HSPs families include HSP70 and HSP90, which work on folding and aggregation of cellular proteins, and regulation of kinetic partitioning between folding, translocation, and assemblage within the cell (Roberts et al., 2010). The mRNA expression of HSPs has been studied in many fish species exposed to various stress conditions such as temperature extremes, pollutants, confinement, handling stress, and acclimatized to salinities (Boone and Vijayan, 2002; Gornati et al., 2004; Tine et al., 2010; Eissa et al., 2017). Consequently, Poltronieri et al. (2007) suggested that HSP70 could be used for evaluating welfare condition in fish. However, the studies on the effect of transport stress on oxidative stress biomarkers and HSPs are limited.

Channel catfish (*Ictalurus punctatus)* is the most numerous catfish species in North America. Nowadays, channel catfish becomes one of the most popular cultured catfish species in China (Xia, 2010). Previous studies demonstrated transport process could cause channel catfish stress (Ellsaesser and Clem, 1986; Refaey et al., 2017). However, no study has reported the changes of antioxidant defense system and hepatic HSPs mRNA expressions in channel catfish suffering from transport stress. Hence, the aim of this study was to investigate the effect of transport on oxidative stress biomarkers and HSPs mRNA expressions, as well as, blood biochemical profiles of channel catfish *Ictalurus punctatus*, and then determine the duration that fish would take to recovery. This study will assist people in understanding the physiological responses of channel catfish suffering from transport stress and improving fish welfare.

## Materials and methods

### Experimental protocol

This study was approved by the Institutional Animal Care and Use Committees (IACUC) of Huazhong Agricultural University (Wuhan, China). We complied by the ARRIVE (Animal Research: Reporting of *in vivo* Experiments) guidelines and Guidelines for Experimental Animals of the Ministry of Science and Technology of P. R. China during the experimental period.

This experiment was carried out in the laboratory of College of Fisheries, Huazhong Agricultural University, Wuhan, China, as described by Refaey et al. (2017). Channel catfish fingerlings were average weight (± SD) 55.57 ± 5.13 g and average length (±SD) 15.98 ± 1.52 cm that acquired from a commercial channel catfish farms (Wuhan, China). Before, the experiment, fish were acclimated for 2 weeks on the laboratory conditions and were fed on a commercial diet until apparent satiation two times daily (10:00 and 19:00). The commercial diet contains 34% crude protein and 3.5% crude fat. Prior to the transport procedure, fish were starved for 24 h.

Before the transport process, fish were subjected to netting, handling and grading, thereafter divided into two groups. One was the control group (the non-transported fish) where fish distributed into seven tanks (at density 20 fish tank^−1^; tank volume 330 L), and were kept in the culture normal conditions. Another group of fish was exposed to the transport stress (the treatment group). Fish in the treatment group were randomly dispersed on five nylon bags (30 L capacity, 50 fish bag^−1^). The bags comprise 10 liters (one-third) of water and two-third of pure oxygen. Next, the bags were placed in a truck and were transported for 3.5 h. After the transport process, fish were distributed into 12 fiberglass tanks (330 L) at the same density in the control group under the culture a normal condition for seven days to determine the recovery time. Fish in both groups were started to eat after 72 h of transport.

### Measurement of water quality

Temperature, dissolved oxygen (DO), pH, and total ammonia nitrogen (TAN) of water were examined and recorded before and after the transport process directly. Water temperature, DO and pH were determined *in situ* by HQ40D Water Analyzer (Hach, Loveland, USA). For TAN, water samples were collected from all the bags before and after transport, and were immediately saved at −20°C until analysis. Water TAN content was assayed using the method of Nessler's reagent spectrophotometry (HJ 535-2009, National Environmental Protection Standard of the People's Republic of China). For the analysis of TAN, water sample was filtered through filter paper (Waterman) and then were added Nessler's reagent (Sinopharm Chemical Reagent Co., Ltd, Shanghai, China) to produce the light reddish brown complex compound. The absorbance of such complex compound is proportional to the ammonia nitrogen contents. TAN content was determined according to the absorbance at the wavelength of 420 nm. Moreover, these parameters were recorded for the control and the treatment groups during the period after transport from 1 day to 7 days (the recovery period). Water quality parameters during the recovery period were remained within acceptable conditions for channel catfish.

### Samples collection

Blood samples were taken at successive time intervals to determine the biochemical and immunological parameters (Table 1) in the blood of fish suffering from transport stress. The endpoint was determined by serum level of cortisol, as well as, other measured biochemical parameters in transported fish recovered to the normal physiological status. Samples of blood were taken before the experiment (the basal level, BL), immediately after packing (AP), and at 0, 1, 6, 24, 72, 168 h after transport. At the time of sampling, fish of each group (*n* = 15/time interval) were randomly, transferred to plastic buckets and were anesthetized in buffer solution with 100 mg L^−1^ tricaine methane sulfonate (MS222, Sigma, St. Louis, Missouri, USA) for 3 min. Blood samples were collected using 2 ml syringes and were pooled in reaction vials (Eppendorf, 1.5 ml). The blood samples were allowed clotting at room temperature for 60 min. Subsequently, serum samples were collected by centrifuge (3000 × g at 4°C for 15 min) and were saved at −80°C until the assay was carried out. For hepatic HSPs mRNA expressions, fish (*n* = 5/time interval) were sacrificed then dissected to obtain the liver. The liver samples were kept at −80°C until analyzed.

**Table 1:** The importance of determination of each parameter on fish welfare.

**Variables**	**Impact on fish care**
Cortisol	An indicator of the primary fish stress response, stimulation of metabolic alterations.
GLU	Biomarker of the secondary fish stress responses, the main source of energy.
TCH and TG	Sources of energy.
TP	An indicator of health status, susceptibility to disease, and innate immunity.
AST	An index of the liver function and status.
T-AOC	Indicator of antioxidant protection against free radical.
T-AOC, GSH-PX, and CAT	Antioxidant system; A kind of detoxification way.
MDA	The indicator of lipid peroxidation; A sensitive diagnostic index of oxidative damage.
NO	Playing an important role in homeostasis, cytotoxicity, cytoprotection, inflammation, protect against oxidative and hypoxia stress.
Hepatic HSPs	Indicators a welfare conditions in fish and immunity fish.

### Serum cortisol assay

Serum cortisol level was examined using a commercial (RIA) kit (Coated Tube Cortisol ^125^I RIA Kit, BNIBT, and Beijing, China) according to the manufacturer's instruction. The experiment was carried out in a round bottom polypropylene tube, Total T, NSB, six standard tubes and sample tubes were coded separately, three repetitions per tube. At first, Cor. standard of 0, 10, 50, 100, 200, 500 mg ml ^−1^ were prepared; The total T tube only needs to add 100 μl ^125^I-Cor, the NSB tube is added with 0 ng ml ^−1^ standard 50 μl, ^125^I-Cor 100 μl, distilled water 100 μl; each standard tube is added with the corresponding concentration standard sample 50 μl, and 100 μl distilled water, rabbit anti-Cor. antibody 100 μl; sample tube adds 50 μl serum, ^125^I-Cor 100 μl, 100 μl distilled water, rabbit anti-Cor. antibody 100 μl. all tubes were shacked and then incubate for 45 min in 37°C water bath. Except the total T tube, 500 μl of donkey-anti-rabbit immune separating agent was added to each tube. After shaking, the tubes were left at room temperature for 15 min and then were centrifuged at 3500 × g for 15 min, discard the supernatant. The sediment of each tube was measured by a Gamma radioimmunoassay counter (GC-911). Cortisol levels in samples were assessed validation by representative parallelism between the sample dilution curve and the standard curve (Li et al., 2012). The intra-assay coefficient is < 10%, and the inter-assay coefficient is < 15%.

### Serum biochemical assay

Serum biochemistry (*n* = 5/time interval) were measured by the automatic biochemical analyzer (Sysmex-800, Sysmex Corporation, Kobe, Japan) using a commercial kit produced by Sysmex Wuxi Co., Let., China. The concentration of serum glucose (GLU) was measured by the hexokinase method. Total cholesterol (TCH) and triglyceride (TG) were tested by the COD-POD method and the glycerol lipase oxidase (GPO-PAP) method, respectively. Serum total protein level (TP) was determined using Biuret methods. Activity of aspartate aminotransferase (AST) was analyzed by the MDH-UV method.

### Antioxidant capacity and antioxidant enzymes activity

Total antioxidant capacity (T-AOC, Kit Serial No: A015), activities of glutathione peroxidase (GSH-PX, Kit Serial No: A005) and catalase (CAT, Kit Serial No: A007-2), and the concentrations of malonaldehyde (MDA, Kit Serial No: A003) and nitric oxide (NO, Kit Serial No: A013-2) were measured by the colorimetric method using the commercial kits produced by Nanjing Jiancheng Bioengineering Institute (Nanjing, China) according to the manufacturer's instruction. These parameters (*n* = 5/time interval) were analyzed using NanoQuant, infinite M200, Tecan.

### Determination of hepatic HSPs mRNA expressions

#### Extraction of RNA and reverse transcription

Fifty milligrams of liver tissues (*n* = 4/time) were homogenized with 1 ml TRIzol® RNA isolation reagent (Takara, Japan) to extracted total RNA according to the manufacturer's guidance. Extracted RNA was resolved in 50 μl RNase-free water. Total RNA was checked by agarose gel electrophoresis, and then using a UV–Vis spectrophotometer (NANODROP 2000c; Thermo Scientific, USA) to determine the quality of RNA. Additionally, the quantity and quality of RNA were estimated by NanoDrop 2000 (Thermo Scientific, Waltham, MA, USA). For gene cloning and real-time RT-PCR, 1 μg of total RNA was used for reverse transcription with a PrimeScript® RT reagent Kit with gDNA Eraser (Perfect Real Time; Takara) in a final reaction volume of 20 μl. The cDNA was stored at −20°C for later use.

#### Real-time RT-qPCR for hepatic HSPs

The primers were designed by using Primer Premier 5.0 software based on channel catfish sequences deposited in the NCBI GenBank (https://www.ncbi.nlm.nih.gov/genbank/). The primers used for gene expression investigation are shown in Table 2. The primers efficiency were estimated using PCR protocol: preheating at 95°C 3 min, followed by 40 cycles of 95°C for 30 s, 60°C for 30 s, 72°C for 40 s, with a final extension at 72°C for 10 min. Next, the PCR products were subjected to electrophoresis in 1% (w/v) agarose gel (1 × TAE) using Andy Safe TMDNA GelStain and were photographed on the Tanon 1,600 gel imager.

**Table 2:** The primer sequences for genes in channel catfish used in quantitative real-time PCR.

**Target gene**	**Sequence (5'−3')**	**PCR product length**	**Accession number**
*18sRNA* F	GGAAAGGATTGACAGATTGATAGC	169	NC030416.1
R	GCCCTCTAAGAAGTTGGACGC	
*Hsc70* F	CAAGATCAGTGACGAGGACAAG	134	XM017489684.1
R	GGTTACAGACTTTCTCCAGTTCC	
*Hsp70* F	CTTGATGTTACCCCTCTGTCTCT	119	NM001200273.1
R	TCAGAGTAGGTGGTGAAAGTCTG	
*Hsp90* F	ATCTGAAGGAGGATCAGACAGAG	112	NM001329313.1
R	CGCTCCTTCTCTACAAAGAGTGT	

Real-time PCR reactions of 25 μl were performed (*n* = 4/time) with 12.5 μl SYBR® Premix Ex Taq II (Tli RNaseH Plus; 2x; Perfect Real; TaKaRa), 2 μl of 5-fold reverse transcription sample, 1.0 μl of each primer (20 mm), and 9.5 μl dH2O. Amplification conditions were as follows: 30 s at 95°C, followed by 40 cycles of 5 s at 95°C, 30 s at 60°C, and 30 s at 72°C. Reactions for the reference gene 18 s were included on each plate. A negative control without cDNA was included in each assay. The results were normalized by 18 s and relative gene quantification was performed using the 2^−ΔΔ*ct*^ method (Livak and Schmittgen, 2001).

### Statistical analysis

Water quality, blood biochemistry, oxidative stress biomarkers, and hepatic HSPs mRNA expressions were tested using SAS (version 9.2). The differences between the groups in each time intervals were examined by *t*-test. While the differences among each time intervals within the group were tested to the one-way analysis of variance (ANOVA) followed by Duncan's *post-hoc* test. Differences were investigated statistically significant at *P* < 0.05.

## Results

### Water quality

Water temperature was ranged from 17.63 ± 0.26 °C before transport to 18.38 ± 0.22°C after transport. DO was significantly declined after transport (2.66 ± 0.46 mg L^−1^) by 3.23 fold compared to before transport (8.60 ± 1.61 mg L^−1^; *P* < 0.05). Further, pH after transport (6.79 ± 0.03) was significantly lower than that before transport (7.53 ± 0.13). However, TAN significantly increased after transport compared with before transport from 0.17 ± 0.07 to 4.06 ± 0.12 mg L^−1^, respectively (*P* < 0.05).

### Serum cortisol concentration

**Figure 1:**
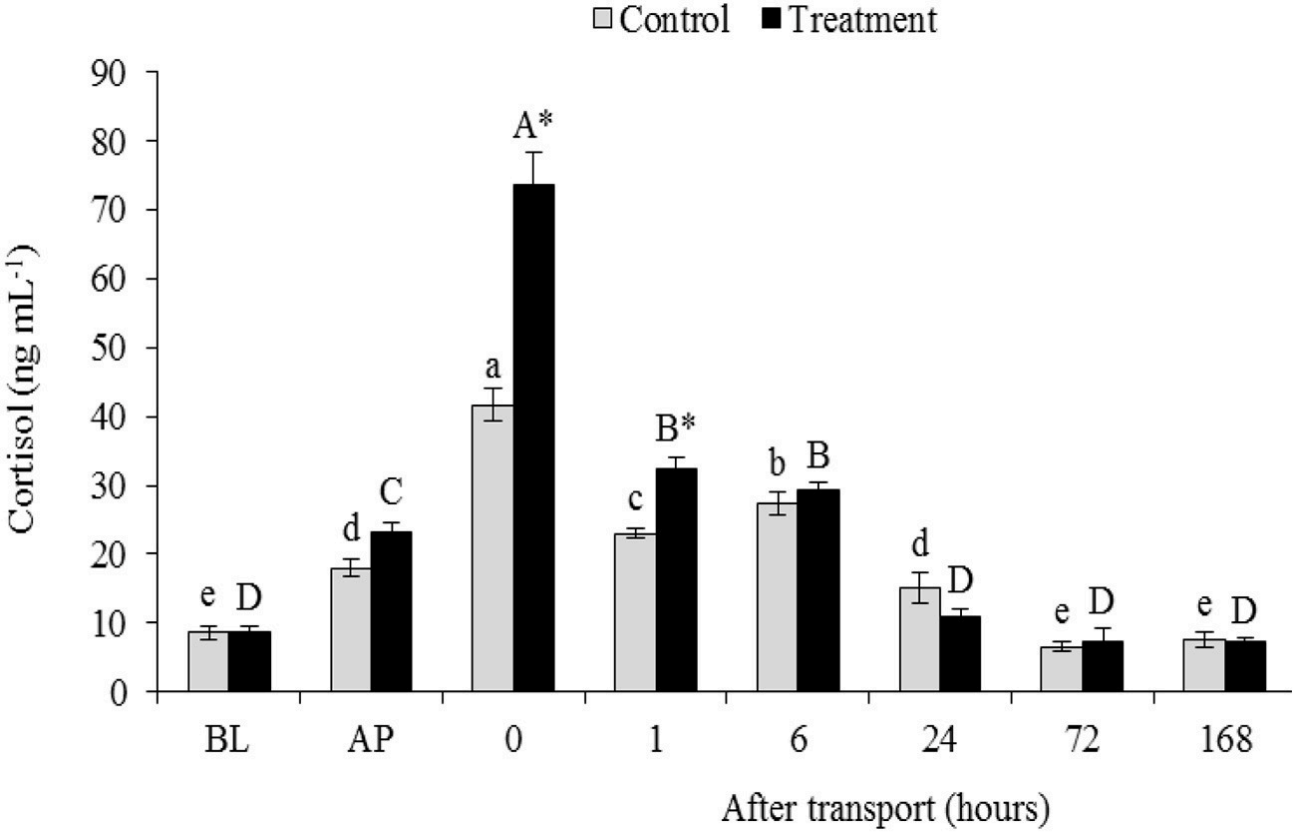
Serum cortisol level of channel catfish sampled at different time intervals after transport (Refaey et al., 2017, new data supplied). Vertical bars indicate standard error; means with an asterisk indicate significant differences (*P* < 0.05) between groups at each time interval. While, capital and small letters indicate significant differences among times intervals within the treatment and control groups, respectively. BL, basal level; AP, after packing; Control, the non-transported fish; Treatment, the transported fish.

Compared to the basal level, the packing process resulted in a significant elevation in serum cortisol concentration either in the control or in the treatment. In both groups, serum cortisol level was gradually increased after packing until reached the peak at 0 h after transport compared with the cortisol level before packing (*P* < 0.05; Figure 1). And thereafter, the cortisol level returned to the BL within 24 h after transport recovery. Serum cortisol concentration in the transported fish was significantly higher than that in the non-transported fish, especially at 0 and 1 h after transport (*P* < 0.05). But, there are no significant difference in serum cortisol concentration between two groups at 6, 24, 72, and 168 h after transport (*P* > 0.05).

### Blood biochemical parameters

**Figure 2:**
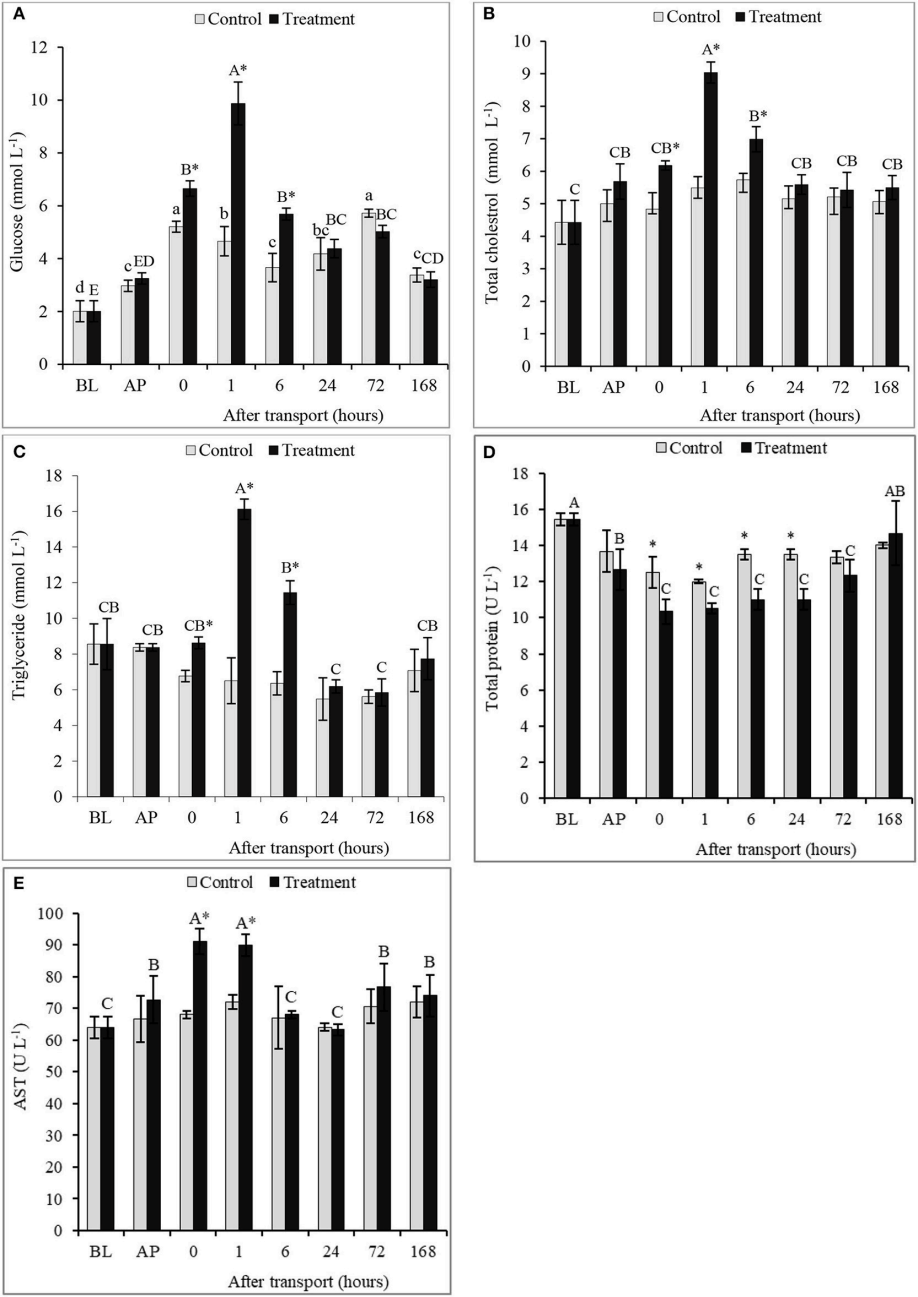
Blood biochemistry profiles **(A)** glucose, **(B)** total cholesterol, **(C)** triglyceride, **(D)** total protein, and **(E)** AST of channel catfish sampled at different time intervals after transport. Vertical bars indicate standard error; means with asterisk indicate significant differences (*P* < 0.05) between groups at each time interval. While, capital and small letters indicate significant differences among times intervals within the treatment and control groups, respectively. BL, basal level; AP, after packing; Control, the non-transported fish; Treatment, the transported fish.

The serum concentrations of GLU, TCH, and TG significantly augmented in the transported fish after 0, 1, and 6 h transport compared to other times of transport and the control group (*P* < 0.05; Figures 2A–C). Compared to the non-transported fish, the highest values of serum GLU, TCH, and TG were recorded at 1 h after transport by 111.9, 64.56, and 147.8%, respectively. However, the contents of GLU, TCH and TG at 24, 72, and 168 h after transport did not differ between the tested groups (*P* > 0.05), and then returned to the BL at 168 h after transport. For the control group, there were no significant differences among time intervals in serum TCH and TG levels (*P* < 0.05). A significant difference in TP content between the control and the treatment was observed (*P* < 0.05; Figure 2D). The transported fish obtained the lowest value compared with non-transported fish at 0, 1, 6, and 24 h after transport. However, no significant difference in TP occurred between groups at 72 and 168 h after transport, as well as, among time intervals in the control group. Regarding AST, the results revealed were gradually increased AP until reached the peak after transport directly and 1 h, thereafter reduced again after 6 h of transport (*P* < 0.05; Figure 2E). The AST level was not affected by the transport process at 6, 24, 72, and 168 h after transport (*P* > 0.05).

### Antioxidant capacity and antioxidant enzymes activity

**Figure 3:**
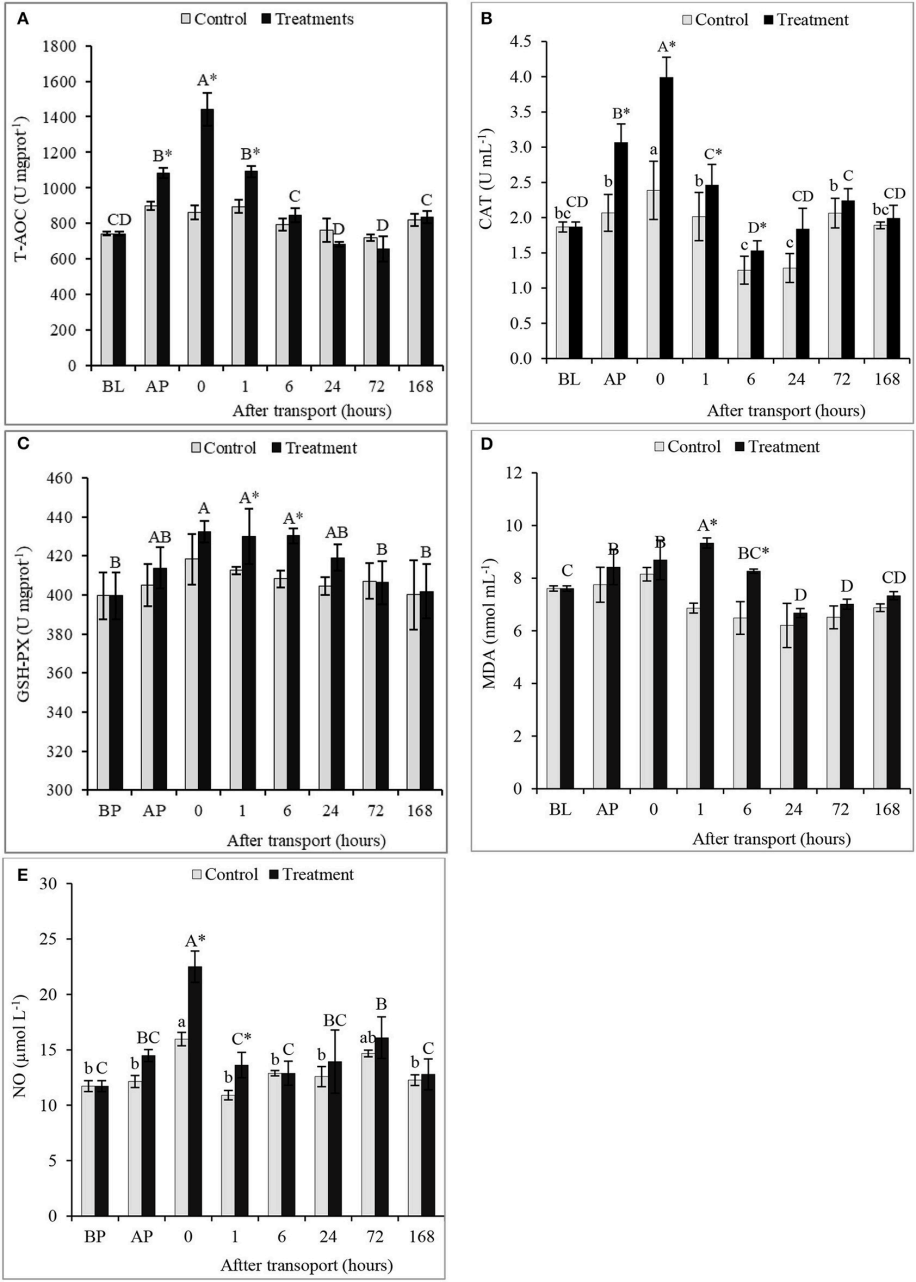
Total antioxidant capacity (T-AOC) **(A)**, antioxidant enzymes activity (as GSH-PX **(B)**, CAT **(C)**) and the concentrations of MDA **(D)** and NO **(E)** of the channel catfish sampled at different time intervals after transport. Vertical bars indicate standard error; means with asterisk indicate significant difference between groups in each time (*P* < 0.05). While, capital and small letters indicate significant differences among times intervals within the treatment and control groups, respectively. BL, basal level; AP, after packing; Control, the non-transported fish; Treatment, the transported fish.

There were significant differences in T-AOC, activities of CAT and GSH-PX, and the concentrations of MDA and NO between the non-transported and the transported fish, as well as, among time intervals in the treatment group (*P* < 0.05; Figures 3A–E). Compared with non-transported fish, a significant increase in T-AOC occurred in the transported fish especially after packing and at 0 and 1 h after transport (*P* < 0.05; Figure 3A). However, there are no significant changes among time intervals in the control group and between two groups at 6, 24, 72, and 168 h after transport (*P* > 0.05). Transported fish showed a significant higher activity of CAT than non-transported fish at AP and at 0, 1, and 6 h after transport (*P* < 0.05; Figure 3B). Although the CAT activity maintained higher in the treatment group at 24, 72, and 168 h after transport, no significant increment was found (*P* > 0.05).

The transported fish exhibited the significant highest activity of GSH-PX and the concentration of MDA at 1 and 6 h after transport and among time intervals of the treatment group (*P* < 0.05; Figures 3C,D). The same trend observed at other time intervals after transport 0, 24, 72, and 168 h without significant difference between groups (*P* > 0.05). However, the content of MDA decreased in transported and non-transported fish compared to the BL in time intervals 24, 72, and 168 h after transport. The differences in GSH-PX activity and MDA content among time intervals of the control group were insignificant (*P* > 0.05). NO concentration of transported fish was recorded the highest values at directly and 1 h after transport by rate 40.89 and 24.91%, respectively, compared to the non-transported fish (*P* < 0.05; Figure 3E). But, the difference in NO contents between groups in time intervals 6, 24, 72, and 168 h after transport were insignificant (*P* > 0.05).

### Hepatic HSPs mRNA expressions

**Figure 4:**
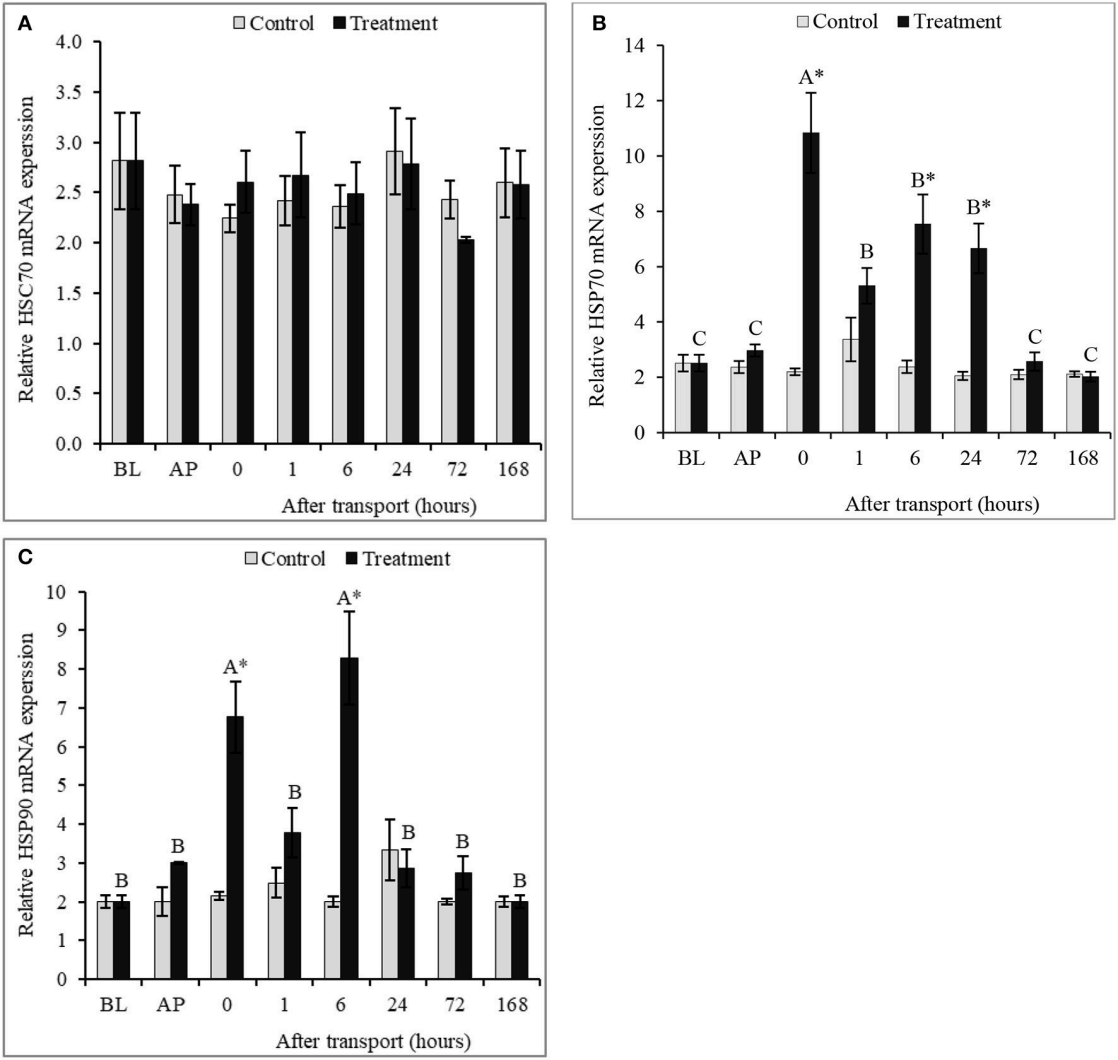
Qualitative expression analysis of hepatic HSPs (HSC70 **(A)**, HSP70 **(B)** and HSP90 **(C)**) mRNA in channel catfish sampled at different time intervals after transport. Vertical bars indicate standard error; means with asterisk indicate significant difference between groups in each time (*P* < 0.05). While, capital letter indicate significant differences among times intervals within the treatment group. BP, before packing; AP, after packing; Control, the non-transported fish; Treatment, the transported fish.

No significant differences were recorded in hepatic HSC70 mRNA expression between groups in all time intervals, as well as, among time intervals within the control group of hepatic HSC70, HSP70, and HSP90 mRNA expressions (*P* > 0.05; Figures 4A–C). Conversely, significant differences in mRNA expressions of HSP70 and HSP90 in liver between the control and the transported fish were detected (*P* < 0.05; Figures 4B,C). Transported fish exhibited significantly higher hepatic HSP70 mRNA expression than the non-transported fish at 0, 6, and 24 h after transport (*P* > 0.05). The expression of HSP70 mRNA has no significant differences between groups at 72 and 168 h after transport (*P* > 0.05). Transport caused a significantly increased in HSP90 mRNA expression at intervals 0 and 6 h after transport. HSP90 mRNA expression between groups was not influenced by transport at time interval 1, 24, 72, and 168 h after transport (*P* > 0.05).

## Discussion

In this study, the transport process caused the deterioration of water conditions such as reducing the DO level, lowering pH, and increasing the concentration of TAN. High stocking density and increased motor activity of fish during the transport period led to an increment both the respiration rate and excretion of nitrogenous waste (Paterson et al., 2003; Gatica et al., 2008). Augmentation of respiration rate resulted in consuming much more dissolved oxygen and augmented the emission of carbon dioxide in the transport packages that reflected on the drop of the DO and pH levels. Besides, the excretion of nitrogenous wastes could increase the level of ammonia in the transport medium that considered one of the main inducers of stress (Paterson et al., 2003). Furthermore, these changes in water quality are stressful for fish, triggering several physiological alterations such as metabolic disturbance, reduced ability to homeostasis, and enzymatic dysfunction (Ruane et al., 2002; North et al., 2006). Similar results obtained in numerous studies on other fish species (Paterson et al., 2003; Dhanasiri et al., 2013; Pakhira et al., 2015).

Fish are often exposed to stressors such as handling, confinement, deteriorating water conditions, and crowding environment under transport conditions (EFSA, 2004). Consequently, transport usually stimulates fish to excrete much more cortisol that is considered as a stress indicator (Barton and Iwama, 1991). In this study, serum cortisol level rose significantly in both the control and the treatment after packing. This suggests that packing procedure could result in fish stress although no fish was transported after packing. Nevertheless, the stress resulting from the packing process was not as high as the transport process stress because the packing process did not take much time. Furthermore, the packing and transport processes significantly augmented the cortisol level, indicating the increased stress level in fish after packing and during the transport. The elevation of cortisol level caused physiological alterations in channel catfish as secondary responses of transport stress with the increase in the contents of glucose, TCH and TG. These parameters related to inducing gluconeogenesis and glycogenolysis pathways, suggesting the energy demand generated by the transport stress (Iwama et al., 1999). Stressed fish activates the metabolic pathway of lipid and triglycerides metabolism to cope with increment energy demand (Montero et al., 1999).

The change in serum protein content is a physiological response that use as indicators of fish health (Tahmasebi-Kohyani et al., 2012). Negative impacts of transport stress on serum TP detected in several of fish species e.g., common carp, *Cyprinus carpio* L. (Dobšikova et al., 2009) and rohu, *Labeo rohita* (Pakhira et al., 2015). Similar results observed in our study. Reduced serum TP may be result of modifying rates of protein synthesis in liver under stress condition. The liver is the chief organ in the synthesis and export of serum proteins (Wright and Anderson, 2001). Additionally, Jackim and La Roche (1973) revealed that the exposure of fish to lack of oxygen causes decrease in protein synthesis. Smith et al. (1996) displayed that protein synthesis in the liver decreased at a rate of more than 90% when exposed crucian carp anoxia. This result confirmed with the outcomes obtained on AST, which indicated the transport triggered the increment of AST. The elevation of AST demonstrates the impairment of normal liver function. Therefore, AST was used as the index of hepatocellular damage (O'Brien et al., 2000).

In aquatic animals, the generation of ROS activates oxidative stress pathways in tissues (Romero et al., 2007) and the subsequent changes in antioxidants enzymes activity. Consequently, the antioxidant profiles were often used as biomarkers of oxidative stress (Kumari et al., 2014). This study showed that transport stress stimulated the antioxidative defense system of channel catfish. Increased antioxidant enzymes activities considered a physiological response to the elimination of ROS generation. GSH-PX and CAT are involved in the reaction of removal of H_2_O_2_ (Cohen and Doherty, 1987; Paital et al., 2011). Compared with non-transported fish and normal fish, the enhancement of T-AOC value in transported fish probably impute to the increment of the enzymatic and/or non-enzymatic antioxidant activity stimulated by transport stress. Similarly, Tian et al. (2016) observed that exposure of yellow catfish (*Pelteobagrus fulvidraco*) to transport stress led to increase of the activities of GSH-PX and T-AOC within 6 h after transport.

Most stressful situation causes a quick increase in lipid peroxidation (Lushchak and Bagnyukova, 2006), leading to induce by oxidative stress (Christia and Costa, 1984). Lipid peroxidation processes produce MDA as a final product. Increased MDA constitutes direct guide of toxic activity resulted by free radicals (Doyotte et al., 1997). Hence, MDA is used as an indicator of cellular ROS and a sign of cellular injuries (Christia and Costa, 1984). The rise of MDA content in transported fish in this study indicated the higher level of lipid peroxidation caused by oxidative stress. This result is in agreement with the increased GSH-PX activity that elucidating the vital role of GSH-PX to reduce lipid peroxidation. However, Tian et al. (2016) stated that transport stress did not effect on peroxidase (POD) activity in yellow catfish.

Nitric oxide helps to increase ability of fish on acclimations to numerous stresses (Malyshev and Manukhina, 1998; Choudhury and Saha, 2012). Transport stress produced the increase of NO content in fish, which probably led to the enhancement of the ability to cope with stress conditions. NO has many helpful effects involving the regain of glomerular filtration and renal blood influx during abnormal physiological situations (Ito et al., 2004), keeping blood pressure (Milsom et al., 1999), as well as, assisting in gas exchange, nitrogen excretion, osmotic and ion regulation (Zaccone et al., 2006). These positive effects could play a major role in increasing oxygenated blood flow to main organs to meet the growing demand for oxygen and energy under transport stress.

In pervious study, Poltronieri et al. (2007) indicated that the expression of HSP70 elevated in sea bass *Dicentrarchus labrax* after transport, suggesting HSP70 can be utilized as a biomarker to transport stress. The same trend observed in this study, transport stress led to the increasing of the mRNA expressions of HSP70 and HSP90 in catfish liver. The increment in HSPs may reflect the improved ability of channel catfish to adapt to transport stress. HSPs play a role in protein misfolding correction and defensive immature polypeptides from assemblage under stress, as well as, protecting the cells from proteotoxicity repaired protein impairment and facilitating the cell growth until conditions are improved (Barnes et al., 2002; Wang et al., 2003; Multhoff, 2007). Likewise, Poltronieri et al. (2008) found the increasing of HSP70 in several tissues of common carp (*Cyprinus carpio*) and rainbow trout (*Oncorhynchus mykiss*) exposed to transport stress. HSC70 is one of the constitutive members in HSP70 family, playing a significant chaperoning role in unstressed cells (Yamashita et al., 2004). Given the results obtained in this study, it's inferred that transport did not significantly affect the expression of hepatic HSC70 of channel catfish. The same trend observed in previous studies on other fish species such as sea bass, common carp, and rainbow trout (Poltronieri et al., 2007, 2008). It is concluded that transport as a stress might not elevate the expression of HSC70 but increase the expressions of HSP70 and HSP90 in fish.

Generally, blood biochemistry profiles, oxidative stress biomarker, and hepatic HSPs mRNA expression did not show any differences between groups after 24 h transport. Besides, these parameters values returned to the basal levels after 72 to 168 h of transport, suggesting channel catfish were not able to reestablish their homeostatic status rapidly under transport stress. Conversely, Urbinati et al. (2004) and Tian et al. (2016) concluded that the matrinxa (*Brycon cephalus*) and yellow catfish needed 24 and 72 h to return to the normal status after transport stress. Schreck et al. (1997) stated that physiological recovery might take 10 to 14 days, depending on the degree of stress. The different results of physiological recovery under transport stress obtained in previous studies probably due to fish species, the physiological responses to fish, transport duration, the degree of stress, and transport condition.

It is concluded that channel catfish is more sensitive to transport condition. The transport process caused the stress of channel catfish, resulting in obvious changes in blood biochemical parameters, antioxidant status, and hepatic HSPs mRNA expressions. The deleterious effects of transport stress on fish physiological function were reversible. A certain recovery period could be required for channel catfish to reestablish homeostasis and recover from transport stress. *I. punctatus* needs to be taken care of at high welfare level after transport for rapid recovery.

## Author contributions

DL and MR designed the experiment. MR performed the experiment, analyzed data, and wrote the manuscript. DL polished the manuscript. All authors reviewed the manuscript.

### Conflict of interest statement

The authors declare that the research was conducted in the absence of any commercial or financial relationships that could be construed as a potential conflict of interest.
